# A Latent Class Analysis of Bullies, Victims and Aggressive Victims in Chinese Adolescence: Relations with Social and School Adjustments

**DOI:** 10.1371/journal.pone.0095290

**Published:** 2014-04-16

**Authors:** Aihui Shao, Lichan Liang, Chunyong Yuan, Yufang Bian

**Affiliations:** 1 State Key Laboratory of Cognitive Neuroscience and Learning, Beijing Normal University, Beijing, China; 2 Psychology Department, Shanghai Campus of Nanjing Politics College, Shanghai, China; 3 Center for Collaboration and Innovation in Brain and Learning Sciences, Beijing Normal University, Beijing, China; Xi'an Jiaotong University School of Medicine, China

## Abstract

This study used the latent class analysis (LCA) to identify and classify Chinese adolescent children's aggressive behaviors. It was found that (1) Adolescent children could be divided into four categories: general children, aggressive children, victimized children and aggressive victimized children. (2) There were significant gender differences among the aggressive victimized children, the aggressive children and the general children. Specifically, aggressive victimized children and aggressive children had greater probabilities of being boys; victimized children had equal probabilities of being boys or girls. (3) Significant differences in loneliness, depression, anxiety and academic achievement existed among the aggressive victims, the aggressor, the victims and the general children, in which the aggressive victims scored the worst in all questionaires. (4) As protective factors, peer and teacher supports had important influences on children's aggressive and victimized behaviors. Relative to general children, aggressive victims, aggressive children and victimized children had lower probabilities of receiving peer supports. On the other hand, compared to general children, aggressive victims had lower probabilities of receiving teacher supports; while significant differences in the probability of receiving teacher supports did not exist between aggressive children and victimized children.

## Introduction

Violence and aggressive behaviors in schools are universal problems, and these problems have attracted the attention of numerous researchers. Researchers have found that aggressive behaviors had negative influences for both the aggressors and the victims, and studies have also found that students who attacked or even used guns against their classmates had a common feature: they have long been victimized [1]. This result implies that we should focus more on those children who are both bullies and victims, which could be a special group, named aggressive victims [2], [3]. So far, many researchers have called for systematic studies on the proportions and characteristics of these children [4], [5], as well as their cognitive and behavioral characteristics. However, there were no consistent conclusions about the distinguishing criteria of aggressive or victimized children till now [6], and there were even fewer consistent classification criteria for the particular group of aggressive victimized children. In addition, there is a lack of studies on whether gender differences exist in aggressive victimized children and whether significant differences could be found between aggressive victimized children and pure aggressive, victimized and non-aggressive victimized children. Therefore, the goals of the present study were (1) to classify the aggressive behaviors and victimized behaviors of adolescent children using the latent class analysis (LCA) method based on the person-centered approach; (2) to study the effects of gender, peer and teacher factors on the latent classification; (3) to investigate whether there were significant differences in emotional adaptation and school adjustment among general children, aggressive children, victimized children and aggressive victimized children.

### The Classification Method and Proportion of Aggressive Victims

Classification according to extreme points is an effective technique for discriminating individual differences, and this technique is widely used in studies of aggression, bullying and other peer relationships related behaviors [7]. Anthony D Pellegrini et al (1999) used the demarcation points of being above 0.8 standard deviations away from the average scores of aggression and victimization to divide 5th grade children into aggressive children, victimized children and aggressive victimized children [2]. The aggressive children accounted for 14% of the sample, and aggressive victimized and victimized children accounted for 5% and 18%, respectively. Salmivalli and Nieminen (2001) used a similar but more accurate method to classify children [8]. To be specific, the bullying group was those whose aggression score was one standard deviation higher than the average score and whose victimized score was one standard deviation lower than the average score; the victimized group was those whose victimized score was one standard deviation higher than the average score and whose aggressive score was one standard deviation lower than the average score; the aggressive victimized group were those whose bullying and victimized scores were at least one standard deviation higher than the average score; and the non-bully and victimized group were those whose bully and victimized scores were one standard deviation lower than the average score [8]. Through this classification method, the bullying group accounted for 10.6% of the total number of people, the victimized group was 6.2%, the aggressive victims group accounted for 1.9%, and the control group was 81.3%. The above classification methods, which were based on the raw scores or *z*-scores, were not only dependent on the individual scores of bullying and victimization but also influenced by the variation of peer group aggression and victimization. Therefore, these classification methods may led to inconsistent conclusions. For example, suppose you have two different samples, and the self-report violations scores range from one to five. Assume that, in sample A, the average score of violation is 3, and the standard deviation is 1.5. In sample B, the average score of violation is 3.5, and the standard deviation is 0.75. When one standard deviation was used as the demarcation points, students in sample A who scored 4.3 would not be divided into violation group, while those with the same score in sample B would be divided into violation group [9].

D. Schwartz et al. (2001) summarized the victimization related studies, and the results showed that aggressive victims accounted for 4%–8% of the victimized group, according to different research methods and practical classification standards. Sekol and Farrington (2010) believed that, because of differences in research methods (e.g., self-report, teacher-report), definitions of bullying and the relevant time period (for example, over the last week or the last month), the proportions of those who both bullied others and were bullied by others ranged from 2% to 29% [10]. However, a number of studies consistently suggested that the proportion of aggressive victims was lower than the pure victimized children [5].

In summary, there are not consistent conclusions on whether certain differences exist in the extent, types and proportions of aggressive victims, aggressors and victims. This is partialy due to the different classification methods, which will affect the exploration of characteristics of different types of aggressive and victimized children.

### LCA: A Person-Centered Approach

Using the latent class analysis (LCA) approach, which is based on person-centered, this article tried to identify and classify Chinese adolescent children's aggressive behaviors, and to explore whether there were different characteristics and influencing factors among the aggressive victims as opposed to pure bullies and pure victims. As mentioned above, researchers often used a certain cut-off score to distinguish the victims, bullies and aggressive victims, and this method may show certain problems.

Similar to cluster analysis, LCA identifies potential classes based mainly on the participants' observable response patterns. This method does not depend on the cut-off scores being set in advance but supposes that a potential classification variable determines the categories of individuals. According to the response patterns of participants on all items, LCA can describe different cross-sectional diagrams.

As a classification method, LCA has many advantages. Different from the cluster analysis, LCA is a method based on models or probability, which means that the model can be verified repeatedly using independent samples [11], [12]. In addition, LCA does not need to standardize the variables and can put predictive variables and outcome variables into the model together (for example, covariates and outcome variables). In addition, different from the cluster analysis, LCA can provide statistical fit indices so that you can determine the model fit and the number of latent classes.

### The Social-Emotional and School-Based Problems of Aggressive Victims, Aggressors and Victims

Aggressive behaviors are universal problems during early adolescent interaction. In past decades, lots of researchers have paid attention to the characteristics of aggressors and victims. Social emotional and school adjustmental problems were found on both bullies and victims, and these problems harmed the development of both bullies and victims [13]–[17].

Those children who were bullied were found to be loneliness, depression, anxiety, distress and have poor peer relationship [18]–[23]. Bullies also showed poorer psychosocial functioning than their classmates. They displayed poorer school achievement and well-being, and perceived less social support from teachers [24], [25]. They also exhibited uncooperative toward peers and little anxiety [26], [27]. Studies also found that bully victims tended to interact negatively with their peers, lacked proper social relationships, and had more social relationship problems [28]. Bully-victims demonstrated high levels of both aggression and depression, and they scored low on measures of academic competence, prosocial behavior, self-control, social acceptance, and self-esteem [7], [24]. Unnever's (2005) study concluded that aggressive victims were more likely to suffer from peers' infractions than pure victimized children [29]. Berkowitz (2012) explored the relationships between teacher supports and school safety as well as their effects on victimized children, aggressive children and aggressive victimized children, and the results showed that the aggressive victims tended to report the lowest level of teacher supports and school safety [30].

Based on previous research, we could conclude that all bullies, victims and aggressive victims display emotional and school-based problems, while aggressive victims experience more emotional and social adjustment problems due to characteristics of both bullies and victims. Studies have shown that children who both bullied others and were bullied by others in school tended to be more restless, get angry more easily, act more impulsively [7], [20], be often disliked by peers [2], get less teacher supports [4], be more likely to suffer from peers' physical attacks [29], and more likely to come from abusive and punitive families [7], [20]. They experienced more emotional and social adjustment problems compared to the bullying and victimized children, such as depression, anxiety, loneliness and less confident [4], [20], [27], [31]–[33], Based on such evidence, the current paper predicted that the aggressive victims would adapt worst among all categories.

There are also other studies focusing on the gender differences among different types of children, and they found that these differences were mainly reflected on specific forms of aggression. Boys appeared to display more physical aggression than girls [19], [22], [34], [35] and girls' relational aggression were more common than boys [36]–[39]. About gender differences of bullies, victims and aggressive victims, prior research indicated that relative to girls, boys were more likely to be bullies and aggressive victims [27], [32], [40].

### The Present Study

Peer relationships were of crucial importance during early puberty, and peer groups were the main social contexts for children's interactions [41]. Peer groups at this stage had important influences on shaping and changing individuals' bullying, victimized behaviors and so on [42]. Early adolescent children experienced a series of significant changes in physical and social aspects. They experienced both the rapid physiological maturity and transitions from elementary school to junior high school, during which they deeded to form a new peer group. In order to attain a dominant position in the new group, some children would exhibit aggressive behaviors [43]. In addition, some large international investigations recently found that the frequency and incidences of peer aggression and victimization peaked in the early periods of middle school stages [22].

This study used LCA method to study adolescent children (Grade 7 students) and to explore the potential categories of bullying and victimization. Many studies had shown that there may be gender differences in bullying and victimization [36], [44]. Meanwhile, peer relationship and teacher-student relationships were crucial social contexts for individuals' development [45], and they would have important influences on children's aggressive behaviors, victimized behaviors and other behaviors; therefore, we explored whether gender, peer supports and teacher supports affected the latent classification. On the other hand, we put loneliness, depression, anxiety and academic achievement as the outcome variables in order to examine whether differences existed in the social and school adjustments among different types of children.

## Materials and Methods

### Participants

Data for the present study were drawn from a cross-sequential research project on development of academic performance and mental health in Hangzhou, P. R. China. In order to conduct an accuracy and comprehensive assessment, we used cluster sampling, and invited all the first-year students of junior high school in a district of Hangzhou city to participate in the study. The original sample consisted of 2457 (1300 boys and 1157 girls) students from 8 public schools. The mean age of the children were 12.6 (SD = 0.5). All children were officially the residents of the district.

### Procedure

A series of scales about children's behaviors and adjustments were administered to the children in classrooms. The administration of the investigation was conducted by graduate students and junior high school teachers who received a professional training. All scales and procedures were approved by the Institutional Review Board of the State Key Laboratory of Cognitive Neuroscience and Learning, Beijing Normal University. Written informed consent was obtained from each participant and their parent. The data were collected in the spring of 2009.

### Measures

All the tools used in this study were from the National Children' s Study of China (NCSC) which was conducted by the State Key Laboratory of Cognitive Neuroscience and Learning at Beijing Normal University. The aim of the NCSC was to examine the psychological development of children and adolescents and the relationship between this development with family, school, and individual factors, and standardized tests were one of the most important research contents. The tools of the NCSC were developed or modified by experts of psychology, education, sociology, cognitive neuroscience and other related fields from home and abroad. If the tools were modified from existing nationality versions, the nationality versions were translated from English into Chinese and then back into English and reviewed by a bilingual psychologist. This set of standardized tests meet the real life of Chinese elementary and middle school students, and have been proved to have good internal consistency reliability, test-retest reliability, construct validity, discriminate validity and criterion-related validity. More details about these tools can be found in the Standardized tests of the NCSC [46].

#### Campus Aggression and Bullying Scale

The scale was used to measure the types and frequency of children's aggression and victimization on campus. The scale was adapted from Children and adolescents aggressive behavior scale and Bully/Victim Questionnaire [46], [47]. In the part of aggression, it included 4 items representing physical aggression (e.g., “I attack other people's body”) and relational aggression (e.g., “gossip behind his back when I get angry with someone”). In the part of victimization, it included 4 items representing physical victimization (e.g., “be hit, kicked, pushed, knocked intentionally by others”) and relational victimization (e.g., “be gossiped behind my back by others”). The scale included 8 items. The responders rated each item on a 5-point scale (0 = never, 1 =  1 time, 2 = 2 times, 3 = 3 to 4 times, and 4 = above 5 times), and the higher the score is, the more serious the issue is. Cronbach's α was 0.78 in the present study, and the test-retest coefficient (interval of one month) of this scale was 0.70. The correlation coefficient of aggression score in subscale and the total score in Children and adolescents aggressive behavior scale was 0.90, and the correlation coefficient of bullied score in subscale and the total score in Bully/Victim Questionnaire was 0.97.

#### Children's Loneliness Scale

This scale was modified from Asher et al.'s (1984) Children's Loneliness Scale (CLS) [48]. The CLS is one of the most widely used self-report scales to screen for children's loneliness. It contains 16 items focus on feelings of loneliness and 8 filler items which is unrelated with loneliness. The modified version used in this study was from the NCSC mentioned above [46]. It retained the 16 items related with loneliness and deleted the 8 filler items. The 16 items include four different kinds of items. These items assessed (a) children's feelings of loneliness (e.g., “I am lonely at school”), (b) children's appraisal of their current peer relationships (e.g., I don't have any friends in class), (c) children's perceptions of the degree to which important relationship needs are being met (e.g., “There are no other kids I can go to when I need help at school”), and (d) children's perceptions of their social competence (e.g., “I'm good at working with other children in my class”). Children responded to each item on a 4-point scale (1 = never true about me to 4 = always true about me), and their responses on each item were summed, with higher scores indicating greater loneliness. In the present study, Cronbach's α for the 16-item version was 0.89, and the test-retest coefficient (interval of one month) was 0.75.

#### Depression Scale for Children and Adolescents

Children's depression was measured by administering a Chinese version of the short form of Childhood Depression Inventory (CDI-S). The CDI-S is a 10-item self-report measure of depressive symptoms for school-aged children and adolescents [49], [50]. There are three alternative responses to each item from which the participant must choose the one that best describes her or him in the past 2 weeks. The items center on a given thought, feeling, or behavior associated with depression, including self-deprecation, loneliness, reduced social interest, anhedonia, selfhate, self-blame, sleep disturbance, fatigue, somatic concerns, and reduced appetite. The items were scored 0, 1, or 2 with a higher score indicative of greater depression. The modified version used in this study was from the the NCSC mentioned above [46]. It also has 10 items which are same with CDI-S, these items center on a given thought, feeling, or behavior associated with depression, and it's scoring method are same with CDI-S. In present study, Cronbach's α was 0.77, and the test-retest coefficient (interval of one month) was 0.81.

#### Manifest Anxiety Scale for Children and Adolescents

Children's anxiety was measured by administering a Chinese version of Reynolds & Richnmond's (1978) Revised Children's Manifest Anxiety Scale [51]. This scale is a 37-item self-report questionnaire designed to measure chronic anxiety (28 items) for children and adolescents between Grade 4 and 9, with nine of the items devoted to a social desirability or lie scale. The modified version used in this study was also from the the NCSC mentioned above [46]. The modified version contains 28 items related with anxiety, and all the items contain three specific dimensions of anxiety (physiological, worry/oversensitivity and concentration). Items were scored 0 or 1 (0 = no and 1 =  yes) with a higher score indicative of greater anxiety. Cronbach's α for the scale was 0.82, and the test-retest coefficient (interval of one month) was 0.72 in this study.

#### Peer and Teacher Supports Scale

This scale is a multidimensional self-report measure of perceived social supports from three sources including parents, peers and teachers, and the scale was developed by the NCSC [46]. In the present study, we chose to examine the peer and teacher supports. Peer supports measure peers' attitudes and emotions towards individuals (e.g., “My classmates think I am clever”), and teacher supports measure teachers' attitudes, expectation and emotions towards individuals (e.g., “Teachers like me”). The scale consists of 12 items and provides 4-point Likert-type responses (1 = never true about me to 4 = always true about me). Higher scores indicate that the children perceived more supports. The subscales' Cronbach's α were 0.86 and 0.83 for the peer and teacher supports items, respectively. In this study, there was a high correlation between the two subscales (r = 0.76).

#### Academic Achievement Tests

In the present study, we tested Chinese and mathematics as measures of academic achievement. Chinese and mathematics were the two main subjects that are common in Chinese schools, Grades in Chinese and mathematics have been found to be a valid measure of school academic achievement in Chinese children [52], [53]. The Chinese and mathematics tests used in this study were from the NCSC mentioned above [46]. The tests were designed by a group of experts and experienced teachers. The contents of the Chinese and mathematics tests are referred to foreign mature academic achievement tests, and curriculum standards of Chinese compulsory education stage developed by Ministry of Education of the People's Republic of China. The tests have been proved to have suitable difficulty and discrimination, and good reliability and validity. Maximum scores for Chinese and mathematics were 100. In the present study, scores on each of Chinese and mathematics were summed to form a single index of academic achievement.

### LCA: Data Analysis Strategy

In order to simplify the analysis and interpretation of the latent class results, bullying and victimized items were dichotomized and denoted as 0 when the score of each item was equal to 0, where 0 means “do not agree with this item,” and 1 when the score of each item was equal to or greater than 1, where 1 means “agree with this item.” The LCA results are based on an exploratory analysis, that is, the analysis does not require a prior distribution or structure assumptions in advance about the class. This is similar to factor analysis, which does not require the factor structure data and items' loads to be specified in advance. The LCA model was fitted by completing a series of steps, usually starting from the 1 classification model and then gradually increasing the number of potential classifications until the model did not show further improvements. The models for this study were performed using Mplus Version 6.1 [11].

For the LCA model, there is not a statistically significant indicator of good model fit. For this reason, a combination of statistical indicators are used to decide the best-fitting model: AIC, BIC, and ABIC. The model that yields the smallest values of these indices indicates the best-fitting model. Additionally, likelihood-based tests are used for model comparison (e.g., chi-squared difference test). Because the common likelihood ratio test cannot be used to test LCA models [54], another index, the BLRT (Bootstrap Likelihood Ratio Test) was used. Recent simulation studies suggest that the BLRT and BIC indices provide the most reliable indicators of the optimum number of classes [55]. Therefore, the BLRT and BIC indicators were mainly used to determine the number of latent classes in this study.

## Results

First, we conducted the latent class analysis for the aggressive and victimized results. Second, we added the covariates (gender, peer supports and teacher supports) into the model and tested for differences of the latent classes in these variables. Third, in order to establish predictive validity, we explored the differences of the different latent classes on loneliness, depression, anxiety and academic achievement.

### Basic Latent Class Analysis

LCA models were run by first testing a one-class model and then exploring models with more classes. Table 1 includes the fit information (i.e., AIC, BIC, ABIC, and *p*-values for the BLRT) for the LCA models with one through five classes.

**Table 1:** Fit Indices for LCA Models with 1–5 Classes.

No. of classes	AIC	BIC	ABIC	BLRT
1	22128.22	22174.65	22149.24	N/A
2	20187.06	20285.74	20231.72	*p*<.001
3	19891.78	20042.69	19960.08	*p*<.001
4	19711.27	19914.42	19803.21	*p*<.001
5	19607.99	19863.38	19723.58	*p* = .061

*Note*. AIC: Akaike Information Criterion; BIC: Bayesian Information Criterion; ABIC: the sample-size Adjusted BIC; B-LRT: Bootstrapped Likelihood Ratio Test.

**Figure 1:**
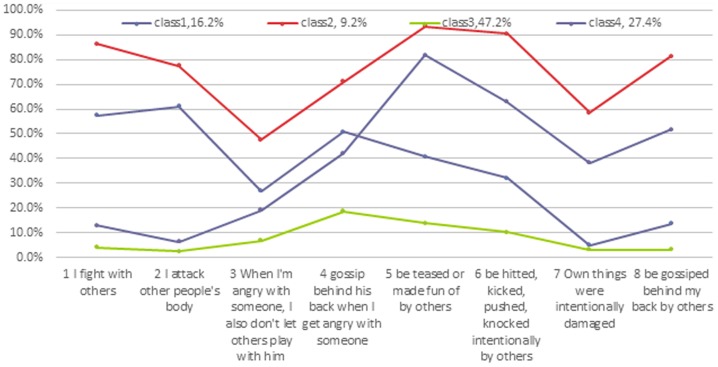
Profile plots for the four-class model. The x-axis is four aggression and four victimization items, and the y-axis the probability of endorsing the items.

The results in Table 1 show that AIC, BIC, and ABIC became smaller as the number of classes increased from 1 to 5, indicating that the model fits better and better. The BLRT results show that significant differences do not exist between the results of Classes 4 and 5, indicating that Class 4 is better than Class 5. Taking into account that the ABIC differences are smaller and the relative simplicity of the model between Classes 4 and 5, the Class 4 model was chose as the best fitting model (results shown in Figure 1: Profile plots for the four-class model).

To interpret the results yielded by LCA, similar to factor analysis, it was important to consider not only the statistical indicators but also the substantive meaning of each of the classes. The conditional item probabilities plots for the four-class model for data on aggression and being bullied in Grade 7 are presented in Figure 1. The item probability values are used to differentiate and explain the latent classes. The item probabilities indicate the probability that a member of a given class would endorse the specific item. Figure 1 presents the profile plots with the four aggression and four victimization items along the x-axis and the probability of endorsing the items along the y-axis.

Examining the LCA analysis results from Grade 7, we know that Class 1 includes 16.2% of the students. This class has a moderate response probability on items 1, 2 and 4, and lower or even zero reaction probability on items 5 and 8, and this type was named as the “aggressive group”. Class 2 includes 9.2% of the whole sample and had a high response probability on aggression items and on victimization items, and this type was named as the “aggressive victimized group.” Class 3 had low response probabilities on the 4 aggressive items and 4 victimization items, and this type was named as the “general group”. This group accounted for 47.2% of the whole sample and was the largest group. Class 4 had lower response probabilities on the 4 aggressive items and moderate response probabilities on items 5, 6 and 8, and this category was named the “victimized group”; this group accounted for 27.4% of the whole sample.

We can conclude from the Figure 1 that the aggressive victimized group accounted for the smallest percentage, and the general children accounted for the largest percentage of the newly enrolled students in 7th grade. The remaining 27.4% of students had lower levels of aggression but also had moderate victimization; 16.2% of students had moderate aggression but less victimization.

### The Social and School Adjustments of Aggressive and Victimized Groups

In order to test whether there are significant differences in the levels of loneliness, depression, anxiety and academic achievement of the different aggressive and victimized groups, these variables was included as outcome variables in conducting the latent class analysis. Loneliness, depression, anxiety, and academic performance were put into the equation as the distal outcome variables to test their differences. In order to examine the differences between each latent category on loneliness, depression, anxiety, and academic performance, a Wald Test was used to test each group. The results show that except the aggressive group and the victimized group, all groups exhibited significant differences in loneliness, depression and academic achievement. The loneliness and depression scores show the following relationship: general children < victimized children and aggressive children < aggressive victimized children. For academic achievement, the opposite relationship exists among each group, that is, general children > victimized and aggressive children > aggressive victimized children. For anxiety, significant differences exist among all of the categories, that is, general children < aggressive children < victimized children < aggressive victimized children (see Table 2).

**Table 2:** Difference Tests of Each Latent Category on Each Variable.

	Loneliness	Depression	Anxiety	Academic Achievement
Aggressive Victims	1.708^a^	1.504^a^	1.428^a^	78.436^a^
Aggression	1.495^b^	1.335^b^	1.298^b^	83.802^b^
Victims	1.530^b^	1.363^b^	1.346^c^	82.787^b^
General Children	1.387^c^	1.239^c^	1.223^d^	85.541^c^

### The Effects of Gender, Peer Supports and Teacher Supports on Aggressive and Victimized Classifications

In order to investigate the differences of gender, peer supports and teacher supports in different latent classes, these variables were included as covariates in the subsequent analysis. Taking into account that the 4-classification model is the best fitting model, gender, peer supports and teacher supports were put as covariates in the 4-classification model. The regressions of each latent variable to gender, peer supports and teacher supports are presented in Table 3.

**Table 3:** Gender (Boys = 0, Girls = 1), Peer Supports and Teacher Supports as Covariates with the general group as the comparison group.

Class	Variables	Legit	SE	t
Aggressive victimized	Gender	−1.299	0.202	−6.439^**^
	Peer Supports	−.133	.030	−4.489^**^
	Teacher Supports	−0.079	0.040	−1.965^*^
Aggressive	Gender	−0.722	0.153	−4.711^**^
	Peer Supports	−0.086	0.026	−3.349^**^
	Teacher Supports	−0.014	0.035	−0.394
Victimized	Gender	−0.193	0.113	−1.705
	Peer Supports	−0.081	0.021	−3.800^**^
	Teacher Supports	−0.023	0.028	−0.836

*Note*. *p<.05; **p<.01.

Table 3 presents the effects of gender, peer supports and teacher supports on aggression and victimization classifications. Class 3 was taken as the general children and made the following comparisons of the three covariates: (1) the likelihood ratio of aggressive victimized group vs. general group; (2) the likelihood ratio of aggressive group vs. general group; and (3) the likelihood ratio of victimized group vs. general group.

As it is presented in Table 3, significant differences existed in gender, peer supports and teacher supports for the probability of belonging to the aggressive victimized group versus general children. Significant differences in gender and peer supports also existed for the probability of belonging to the aggressive group versus general children. A significant difference in the probability of belonging to the victimized group and general group only existed for peer supports.

## Discussion

Using LCA method, this study analyzed the classifications of aggressive and victimized behaviors of junior high school during early puberty and also explored the influences of gender, peer supports and teacher supports on the classification, as well as different types of children's emotional and school adaptations. The results showed that the four-class model best represented the aggressive and victimized conditions of adolescent children, which includes general children, aggressive children, victimized children and aggressive victimized children. The four categories significant differed from each other in terms of loneliness, depression, anxiety and academic achievement, among which the aggressive victims adapted the worst. This study further found gender, peer supports and teacher supports had significant effects on the four categories.

### The Percentages of Aggressors, Victims and Aggressive Victims

Children's self-report aggression and victimization levels were classified using LCA. For newly enrolled Grade 7 students, nearly half of the children (47.2%) had lower levels of bullying and victimization and were not involved in bullying and victimized affairs. However, more than half of the remaining children were involved in bullying and victimization affairs, and 27.4% of students had a lower level of bullying but are moderately victimized, while 16.2% of students have a moderate level of bullying but are rarely victimized. The aggressive victimized children accounted for only 9.2% of the total number of students, which accounted for the smallest percentage. The proportions of students involved in aggressive and victimization affairs were higher than the results of classification based on raw scores or *z* scores. As described above, past classifications methods about aggression and victimization often used extreme scores (e.g., *z* scores); however, due to limitations of the z-score method itself, different research conclusions were not consistent with the proportion of aggressive children [5], [10]. If the method of raw scores or standard scores was adopted, the percentage of aggressive and victimized children in the total number would be artificially restricted. This method is a relatively subjective grouping method, and subsequent analyses could not really reflect the characteristics and proportions of these groups. The latent class analysis is an exploratory, empirically-driven method, and it does not require diversified model limitations; therefore, this method can often best describe the data. The percentages of aggressors, victims and aggressive victims in this study were roughly consistent with previous conclusions [5], [10].

### Emotion and School Adjustments of Different Categories

Research results indicate that, relative to other categories, those children who scored low on both aggression and victimization items (the general group) rarely felt loneliness, depression, anxiety and other negative emotions, and they also had better academic achievement and better emotional and school adjustments. There were no significant differences between aggressive group and victimized groups on loneliness, depression and academic achievements, while the victimized groups experienced more anxiety emotions than the aggressive group. Certain emotional and school adjustments problems existed in the two groups.

Importantly, this study found that aggressive victimized children were the most problematic group among all categories. Children who belonged to this categories tended to have lower academic achievement, feel more lonely, experience more depression and anxiety, and have major emotional and school adaptation problems. This may be because, relative to other children, the aggressive victimized children are often disliked by their peers [2], get less peer assistance and teacher supports [4], and are more likely be attacked by peers [29], which results in a more negative self-perception, poor emotional adjustment and poor academic achievement. The aggressive victimized children often feel insecure in school. These children who bully others (which leads to their being disliked by teachers and peers) are often victims of bullying behaviors, as well as receiving less social support; all these factors lead to such children to become high-risk children.

Similar to previous studies, this study used LCA and found that the aggressive victimized children constituted a special group of a smaller proportion. These special children had poor academic performance, more mental health problems, more peer rejection, and were the most disadvantaged category of bullying and victimization groups. Teachers, parents, and clinicians should pay more attention to these children.

### The Effects of Gender, Peer Supports and Teacher Supports on Latent Categories

Based on previous research, gender was chose as a covariate in order to investigate the effects of gender on classification. This paper found that gender had a significant impact on bullying and victimization types for the newly enrolled junior high school students. Specifically, relative to general children, boys were more likely to become aggressive victimized children or aggressive children; while relative to general children, gender did not have a significant effect on being victimized children. This indicates that, for the newly enrolled junior high school students, boys and girls are being victimized equally, and this result is consistent with some related international studies [56]. This study also found that boys were more likely to become aggressors or aggressive victims, which is affected by community values and gender stereotypic behaviors [36], [57]; boys' bullying behaviors are often compatible with traditional gender norms, and boys are more likely to attack peers to attain social status and control.

A large number of psychological studies showed that peer support and school supports played an important buffering role for disadvantaged children during puberty (buffering effects). As important influence factors for children's development in the adolescent stage, peers and teachers protected children's from aggressive and victimized behaviors [58]–[60]. Studies have shown that, compared to general children, peer supports played an important role for aggressive, victimized and aggressive victimized children, and all of these children had fewer peer supports. Those children who receive fewer peer supports and experience more peer rejection tend to feel more frustration and develop negative attitudes and emotions towards others and themselves, which might lead to subsequent incidences of bullying behaviors. Chen, Huang, Wang, and Chang (2012) did a follow-up study of 9 to 12 year old students of aggressive behavior and peer relationships and found that peer relationships had direct and indirect negative effects on subsequent aggressive behaviors [61]. On the other hand, other children may think that children who lack peer supports and protection are weak [62], [63], which could lead to their further victimized behaviors.

This study also found that teacher supports had inconsistent effects on different types of children. Studies have shown that there were no significant differences in the probabilities of receiving teacher supports among aggressive children, victimized children and general children. However, relative to general children, aggressive victimized children received less teacher supports and help. Consistent with previous studies, current paper found that aggressive victimized children often reported the lowest levels of teacher supports. The aggressive victimized children were more likely to suffer from social isolation and get less social support (peer supports and teacher supports), and teachers were less willing to help them when they are bullied by others [4], [30].

### Limitations

Based on an individual-centered approach, we classified aggressive and victimized groups. This method overcame some of the limitations of traditional classification methods, and this study has certain theoretical and practical significances for the identification and intervention of aggressive and victimized individuals. However, there were still certain limitations of this study. First, this study was a cross-sectional study using only one year data; therefore, it could not investigate changes over time of bullying and victimization behaviors and also could not investigate which factors may affect those developments and changes. Future research should take a follow-up study paradigm in order to explore the developments and changes of bullying and victimized groups over time. Second, only self-report method was used in this study, and it may exist common method biases, which may influence the generalization of the results. Self-reports, peer-nomination, teacher-reports and other data collection methods should be used together in future studies to overcome the biases caused by the self-report method.

## Conclusions

Using latent class analysis, this study found that, (1) based on aggression and victimization, adolescent children could be divided into general children, aggressive children, victimized children and aggressive victimized children. (2) There were significant gender differences in the aggressive victimized children, victimized children and general children. That is, relative to general children, aggressive victimized children and aggressive children were more likely to be boys, and relative to general children, gender differences did not exist for the probability of whether victimized children are boys or girls. (3) There were certain differences in the levels of loneliness, depression, anxiety and academic achievement of the aggressive victimized children, aggressive children, victimized children and general children; the aggressive victimized children were the worst adapted group among the four categories. (4) As protective factors, peer supports and teacher supports had important impacts on bullying and victimized behaviors. Relative to general children, aggressive victimized children, aggressive children and victimized children were less likely to receive peer supports. Relative to general children, aggressive victims were less likely to receive teacher supports, but relative to general children, significant differences did not exist in the probability of receiving teacher supports between aggressive children and victimized children.
